# Letter from C. W. Spalding

**Published:** 1866-02

**Authors:** C. W. Spalding

**Affiliations:** St. Louis


St. Louis, Jan. 1866.

Dear Doctor:—Having failed to comply with your request to prepare an article for your January number, I will make such amends as I can by sending you a dish of promiscuous gossip.

A few weeks of absence makes home appear all the more pleasant on our return, and the changes that occur during that absence, though they may seem of but little moment to one who observes them as they take place, day by day, appear to the absentee, who takes them in all at once, as worthy of more particular notice.

A bridge of ice afforded me an easy transit across the "Father of Waters," and the crushed and sunken steamers and barges, that lay in promiscuous confusion "along shore," told of the power of the great river when obstructed in his onward flow to the ocean.

As a matter of more interest to the readers of the "Register" than the foregoing, I will mention the removal of the dental depot of Dr. A. M. Leslie & Co., to a more central and much more desirable locality than that formerly occupied by that enterprising firm. Their new location is on Chestnut street, between Fifth and Sixth streets, where they have a very fine, large store, well fitted up and very conveniently arranged for the accommodation of their patrons. Their stock of dental instruments and materials is large and full, and when added to a fine stock of surgical instruments and appliances will fill their capacious store to overflowing. Enterprises of this character deserve the patronage and support of the profession. May their success equal their efforts, is the best wish I can make them.

I also notice that Dr. John S. Clarke, formerly of this city, but, of late years, of New Orleans, has returned to his "first love," and opened an office in this city.

I am rejoiced to see the prosperous look which the "Register" puts on with the beginning of the new year, and considerable change which you have made in its title—a decided improvement. Our periodical literature is (to borrow a military phrase) a very important arm of the service, and its influence is too wide spread to allow it to retain anything local in *appearance* even in a name.

In looking over the January number of the "Cosmos," I notice some misapprehension existing in the mind of the editor, relative to a remark of one of the speakers in a discussion which took place at the Chicago meeting last summer. In the discussion on physiology, if I remember rightly, the speaker illustrated his subject by reference to the course pursued by honey bees in replacing a lost queen; and in describing the process used the word *drone* instead of *worker*.

Such a remark would mislead no one who had studied the subject of sexuality among bees, for it would be well known to all such that drones being males could not, of course, be transformed into females; but workers being undeveloped females could, when taken in a larva state, be so fed as to cause a development of the sexual organs, and thus become queens. As I mentioned the matter to the speaker soon after the adjournment, I am the more confident that the editor of the "Cosmos" has fallen into an error.

Hoping soon to be with you again,

I remain very truly yours,

C. W. Spalding.

